# Determination of Endocrine Disrupting Chemicals in Water and Wastewater Samples by Liquid Chromatography-Negative Ion Electrospray Ionization-Tandem Mass Spectrometry

**DOI:** 10.3390/molecules25173906

**Published:** 2020-08-27

**Authors:** Ghada Aborkhees, Renata Raina-Fulton, Ondiveerapan Thirunavokkarasu

**Affiliations:** 1 Department of Chemistry & Biochemistry and Trace Analysis Facility, University of Regina, Regina, SK S4S 0A2, Canada; aborkhees1984@yahoo.com; 2 Environmental Protection Services Section; Water Security Agency, Regina, SK S4P 4K1, Canada; o.tarasu@wsask.ca

**Keywords:** endocrine-disrupting chemicals, solid-phase extraction wastewater, liquid chromatography-negative ion electrospray-tandem mass spectrometry, matrix effect

## Abstract

A liquid chromatography-negative ion electrospray ionization-tandem mass spectrometry method was developed for the simultaneous analysis of bisphenol A, 4-octylphenol, 4-nonylphenol, diethylstilbestrol, 17β-estradiol, estriol, estrone, 17α-ethinylestradiol, prednisone, and prednisolone. This method used solid-phase extraction with an elution solvent of acetonitrile to improve the stability of the analytes. To maintain the stability of analytes analyses were completed within five days. The recoveries ranged from 84 to 112% and the relative standard deviation of analysis of duplicate samples was <10%. The limits of quantitation were 1–10 ng/L. Surface water and wastewater were obtained from five wastewater treatment plants in Saskatchewan. Matrix effects were moderate to severe. Using standard addition calibration, all analytes except diethylstilbestrol and 17α-ethinyl estradiol were detected. There was a low frequency of detection of the target analytes in upstream and downstream water, indicating good removal efficiency during the wastewater treatment process. Bisphenol A and 4-nonylphenol were the only analytes detected downstream. Bisphenol A was the most frequently detected in raw wastewater (133 to 403 ng/L). Estriol was detected more often in raw wastewater than estrone or 17β-estradiol. This is the first Canadian study with the detection of prednisone and prednisolone with concentrations at 198–350 ng/L in raw wastewater at 60% of the wastewater treatment plants.

## 1. Introduction

Endocrine-disrupting chemicals (EDCs) are a group of chemicals or mixtures of chemicals in the environment (air, soil, or water supply), food sources, personal care products, and manufactured products that exert hormonal-mimicking effects and interfere with the hormonal regulatory mechanisms of living organisms. The occurrence of EDCs in wastewater, which is released into aquatic environments, is of particular concern because of their potential impact of wildlife as their persistence in water supplies for humans. A variety of methods have been used for targeted analyses of endrocrine-disrupting chemicals (EDCs) in surface and wastewater, and often focus on selective groups or a limited target list of EDCs to address the need to optimize the separation and mass spectrometric detection (MS), and to ensure stability and good recoveries of the EDCs in sample preparation procedures. Often estrogenic EDCs are analyzed separately from non-estrogenic EDCs [1,2,3,4,5,6,7], and most existing methods can meet detection limits required for monitoring requirements but are designed for specific sample matrices. Selective estrogenic EDCs are included in the watch list for the European Union and the United States Environmental Protection Agency [8,9]. This work focuses on natural estrogens (17β-estradiol, estrone, and estriol), synthetic estrogens (17α-ethinylestradiol, and diethylstilbestrol (not prescribed since 1971 in Canada and United States and was previously prescribed to prevent miscarriage and premature delivery)), xenoestrogens (bisphenol A, 4-octylphenol, and 4-nonylphenol), and two glucocorticoid drugs (prednisolone and prednisone) that are not routinely analyzed. Natural estrogens enter wastewater from domestic and agricultural discharge, while synthetic estrogens and glucocorticoid drugs are prescribed to patients with human excretion as the main source entering wastewater [4,10,11,12,13,14,15,16]. Prednisone and prednisolone are prescribed to patients within Saskatchewan in both hospital and outpatient settings for a variety of health issues, including arthritis, dermatologic, ophthalmic, respiratory, and gastrointestinal diseases, and cancer treatments [5]. Prednisolone is the metabolite or degradation product of prednisone and is preferred for patients with impaired liver function. Bisphenol A is used in the manufacturing of plastics, rubber, and flame retardants and is released from plastic and polycarbonate materials into wastewater and surface water [10]. Alkylphenols are used as surfactants, plastic additives, and emulsifiers, and major sources include discharge during the manufacturing of plastics and the use of detergents [12,17,18,19,20,21,22,23].

These analytes that are EDCs are typically not included in multiresidue analysis methods due to specialized needs in sample preparation or separation-mass spectrometric detection. Due to the high complexity of the wastewater matrix, a number of alternative methods have been used for analysis of estrogenic and xenoestrogenic compounds. Gas chromatography methods without derivatization have been used for bisphenol A (BPA), octylphenol, and nonylphenol in surface and wastewater with recoveries ranging from 88–112% and 111–129%, respectively [24,25]. To improve MS sensitivity or peak shape in the separation for these more polar analytes as well as estrone (E1), 17β-estradiol (E2), and 17α-ethinylestradiol (EE), silylation prior to analysis has also been used to produce stable trimethylsilyl or tert-butyldimethylsilyl derivatives with recoveries in the range of 70–120% [26]. However, recoveries achieved for analytes can vary with conditions used in the derivatization, including the presence of matrix, leading to greater challenges in selecting suitable methods for wastewater [27]. More commonly now, liquid chromatography-mass spectrometry is selected to avoid the need to derivatize estrogens or xenoestrogens. Natural hormonal estrogens are analyzed in negative ion mode and can be ionized using atmospheric pressure chemical ionization (APCI), but detection limits are generally lower when using electrospray ionization [6]. Positive ion mode in APCI can be used for estrogens but does not provide adequate sensitivity for xenoestrogens [28,29]. Derivatization has also been used when analyzing estrogens and xenoestrogens in biological fluids and plasma to improve the electrospray ionization in positive ion mode for molecules with a phenolic group [7,30,31], but these methods have not been applied successfully to wastewater. Other derivatization agents, including pentaflurobenzyl bromide and 2-fluoro-1-meyhtlpyridinium p-toluenesulfonate have been used for surface water; however, more MS signal suppression is observed in wastewater samples [32,33,34].

Solid-phase extraction (SPE) has been used for the selective analysis of estrogens generally in cartridge format [29,35,36]; however, when xenoestrogens are included, these methods often require higher elution volumes or stronger solvents [13,37,38] and as such these different groups of analytes are often analyzed in separate methods [39,40,41]. SPE discs with further Florisil SPE cartridges have also been used for the extraction of natural estrogens from surface water [1]. Recoveries have been reported <15% when divinylbenzene (DVB) SPE discs followed by NH2 SPE cartridges were used, with small improvements in recoveries when Florisil clean-up was used [1]. The selection of the SPE sorbent and sorbent capacity is critical in the extraction process with the severity of the matrix effect expected to increase from the surface water to the wastewater. The objective of this work was to develop a reliable and sensitive method for these selective EDCs that does not require derivatization, while still addressing the stability issues of some target analytes, and that can be applied to the highly complex sample matrix of wastewater. The method was designed to address in one analysis a larger range of target analytes that are amenable to electrospray ionization in negative ion mode. This includes the addition of two drugs (prednisone and its metabolite prednisolone) that have not been reported in studies in Canada and are not routinely screened for in water quality studies in many countries.

## 2. Results and Discussion

### 2.1. Optimization of the Solid-Phase Extraction Procedure

**Figure 1:**
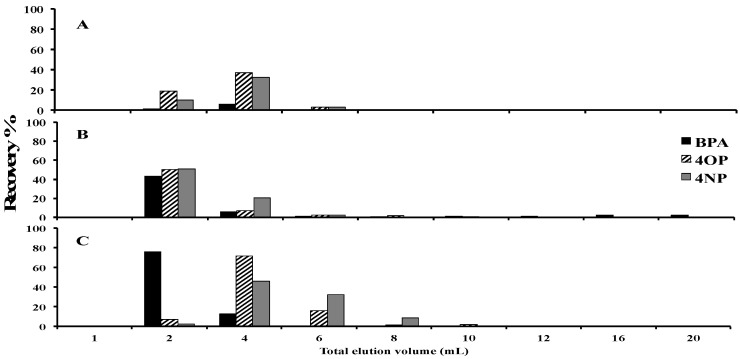
Elution profile of BPA, 4OP and 4NP for SPE with C18 (1000 mg/6 mL) sorbent and total volume of 20 mL of elution solvents. Elution solvent: (**A**), methanol; (**B**), 50/50 *v/v*% methanol/acetonitrile; and (**C**), acetonitrile.

Two solid-phase extraction (SPE) sorbents (Oasis HLB and Oasis MAX) were selected from previously published methods for other pharmaceuticals not included in these studies for evaluation [42,43]. In addition, the C18 SPE sorbent was selected as an alternative sorbent for the evaluation, as SPE could be completed under neutral pH conditions, which reduced or eliminated the need for additives for pH adjustment of water or elution solvents. The addition of additives in solvents used in SPE such as acids (formic acid) or bases (ammonia) to alter pH was found to alter mass spectrometric sensitivity if not remove completely during the pre-concentration step. The pK_a_ of the target analytes is high (pK_a_ for bisphenol A (BPA), estrogens and surfactants, prednisone, and prednisolone were 9.8, 10.2–10.5, 12.4, and 12.5, respectively) such that the target analytes would not be ionized [38,44]. BPA, 4-octylpphenol (4OP), and 4-nonylphenol (4NP) are also the most prone to degradation of the targeted analytes, and as such, these analytes were used for selection of solvents and best sorbent for SPE. After loading of the sample on the SPE sorbents such as C18 or Oasis HLB, a wash step with a volume of 2.0 mL of H_2_O was chosen as higher volumes resulted in earlier elution of the BPA into the F0 wash fraction. As can be seen from Figure 1, the first 1 mL of the elution of acetonitrile showed no detection of target analytes, so ~0.8 mL of acetonitrile was also eluted into the F0 wash fraction to aid in the removal of more polar matrix components. Figure 1 shows that the low recoveries (<80%) for BPA, 4NP, and 4OP were obtained when the elution from C18 was completed with 20 mL of methanol or 50/50 *v/v*% methanol/acetonitrile. Furthermore, BPA, 4OP, and 4NP observed stability issues when the sample preparation took over one week even if the extracts were stored in the refrigerator overnight. To pre-concentrate the target analytes, >30 h was required for the drying of the extracts to 0.8 mL at 0.5 mL/h in the SPE apparatus. Faster drying rates led to the loss of target analytes. The degradation of BPA and 4NP analytes in methanol has been previously reported to occur within seven days [29]. The precipitation of the injected samples occurred in the LC-MS/MS system when methanol was used as the elution solvent for SPE and dilution of samples before LC-MS/MS analysis. This precipitation was evident on the pre-column over time and deterioration in peak shapes was observed. Retention time shifts and reduced MS sensitivity were also observed if samples were stored for more than one week from the time of elution from the SPE cartridge. Consequently, the time to completion of analysis from the elution step was reduced to five days or less, and under these conditions no degradation of the sample was evident. An improved stability of target analytes through the sample preparation procedures was also observed when acetonitrile was used as the elution solvent. BPA eluting within the first 5 mL of acetonitrile, while 4OP and 4NP required 10 mL and 15 mL of acetonitrile to adequately recover the target analytes from the C18 SPE sorbent, respectively (see Figure 1C). Consequently, an elution volume of 15 mL of acetonitrile was chosen to obtain recoveries >80%.

**Figure 2:**
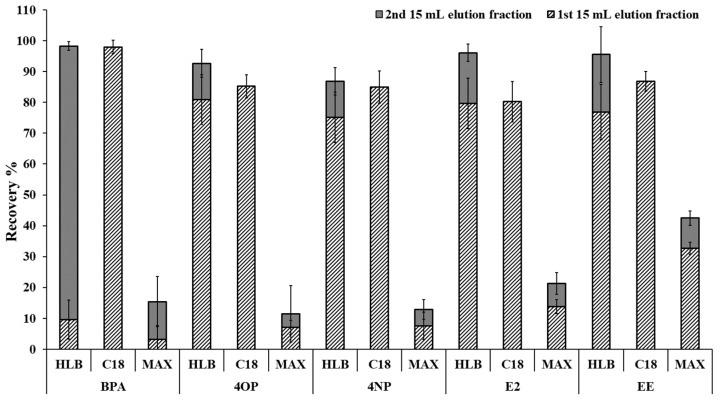
Average recoveries of selected target analytes BPA, 4OP, 4NP, E2, and EE on Oasis HLB and MAX at pH = 3, and C18 at pH = 7. F1, 1st eluted fraction with volume of 15 mL acetonitrile; F2, 2nd eluted fraction with volume of 15 mL acetonitrile. The error bars represent the relative standard deviation determined from triplicate samples.

Recoveries of a larger range of target analytes on the three SPE sorbents (Oasis HLB, Oasis MAX, and C18) were then evaluated. A volume of the F1 fraction (1st eluted fraction where target analytes are detected) was selected to be 15 mL of acetonitrile as this volume was considered the maximum volume practical, as subsequent drying steps required ~30 h (0.5 mL/h drying rate). As noted previously, longer sample preparation times could lead to problems with the stability of the target analytes. To ensure all the target analytes were eluted into the F1 fraction, a second elution with an additional 15 mL of acetonitrile (F2 fraction) was evaluated for the presence of target analytes. When using Oasis MAX SPE sorbent, the elution process also differed with 5 v% NH_4_OH in H_2_O used in the wash step and 2 v% formic acid in acetonitrile, used for the elution following a similar procedure to the previously reported method [44]. Poor recoveries were observed with Oasis MAX and could be partially attributed to the presence of additives (formic acid) used in the elution solvent, which could significantly reduce the MS sensitivity even if only traces of formic acid were present in the injected sample. Oasis HLB provided good recoveries (>80%) for all target analytes but required large elution volumes as analytes were still present in the F2 fraction (see Figure 2), and this would subsequently increase the time required for the drying step. With C18, >80% recoveries of the target analytes were observed in the first fraction, F1. The reproducibility of recoveries for target analytes (obtained with F1 and F2) when C18 SPE sorbent was used was also better than Oasis HLB, as shown in Figure 2. Other studies have also chosen C18 (500–1000 mg) for the analysis of other EDCs not selected in this study due to better reproducibility [45,46].

Recovery tests were completed for the full list of target analytes in this study at three concentrations (20, 100, and 500 ng/L). To account for a small fraction of target analytes observed in the F2 fraction when the C18 sorbent was used (see Figure 2), the elution volume was increased from 15.0 to 15.2 mL. The recoveries of the target analytes, as shown in Table 1, were in the desired range of 85–110% except at the low concentration (20 ng /L) for estriol (E3), 4NP, and (PRNL), but still greater than 80% and acceptable. The percent relative standard deviation was less than 15%.

### 2.2. LC-ESI^-^-MS/MS Conditions

Estrogenic ECDs have relatively weak retention on C18 columns, and additives can lead to diminished MS sensitivity for these target analytes that are ionized in negative ion mode. It was found that Phenomenex Gemini C18 (150 mm × 2.0 mm, 3 μm) with a precolumn, Phenomenex Gemini C18 (4 mm × 2.0 mm), could provide adequate retention of EDCs and sufficient chromatographic resolution for EDCs when methanol/H_2_O was used as the mobile phase. Table 1 shows that all analytes have unique selective reaction monitoring transitions (SRMs). The SRM with the strongest response for the target analytes was selected as the quantitative SRM, while the second most intense response was selected for the confirmation SRM. E3 and glucocorticoids (PRDN and PRNL) were eluted first, followed by BPA. The target analytes, 4OP and 4NP, and the volume check standard, gemfibrozil-d_6,_ were retained longer and needed higher concentrations of methanol in the mobile phase, such that the gradient for the separation ranged from 70 to 100% methanol. Although BPA and diethylstilbestrol (DES), E1, and equilin (Eq) partially co-eluted on a C18 column (see Supplementary Materials, Figure S1), their response could be isolated using unique quantitative and confirmation SRMs.

MS sensitivity of the target analytes, particularly BPA, 4OP, and 4NP were significantly lower when the organic modifier was acetonitrile as compared to methanol. The use of methanol allowed for a higher percentage of organic modifiers to be used in the mobile phase, which also improved MS sensitivity. As noted previously, the extract from the SPE stage contained acetonitrile, and these extracts were diluted in a 50/50 *v/v*% mixture of methanol/acetonitrile just prior to the analysis to minimize degradation while maintaining MS sensitivity for the LC-ESI^-^-MS/MS analysis. Several deuterated standards were evaluated for internal standard, surrogate standard, and volume check standard (extract volume determination). BPA-d_16_ and E1-d_4_, were selected as internal standards as they had no response for the SRMs for the target analytes over the concentration standards range used. Deuterated and non-deuterated standards (BPA and BPAd_16_, E1 and E1d_4_, and 4NP, and 4NPd_4_) co-eluted with each other; however, the response of the SRMs could be isolated from each other. At the concentration of the deuterated standard used, there was no response from these deuterated compounds at the SRM selected for BPA, E1, or 4NP. Other deuterated standards that were evaluated included 17α-estradiol-2,4-d_2_ and 17β-estradiol-2,4-d_2_; however, they were excluded from this study as the co-elution with E2 occurred and the deuterated standards also gave a response for the SRM of E2 at the lowest concentrations feasible for detection.

As shown in Table 1, each analyte had at least one confirmation SRM. In some cases, there was only one fragment of significant abundance with collision-induced dissociation such that [M − H]^−^→[M − H]^−^ was selected for the confirmation with no collision energy (4OP; 205 > 205 and 4NP 219 > 219). For confirmation, the ratio of SRM1/SRM2 must be within the relative standard deviation (RSD) determined from the day of analysis and was within an acceptable relative standard deviation range (RSD 1.39–13.0%). The minimum concentration that showed a percent relative standard deviation (%RSD) of less than 25% from the regression line determined from least-square regression analysis for both the quantitative and confirmation SRM transition was established as the limit of quantitation (LOQs). LOQs were 1.0 to 10 ng/L. Linearity was from LOQ to 100 ng/L with regression coefficients >0.990 for all the analytes (see Supplementary Materials, Table S1). This concentration range was appropriate for the determination of the target analytes in the extracts from SPE. The analysis of blank samples completed before and after each set of samples or standards showed no carry-over problems.

Various mobile phase additives such as formic acid and ammonia were evaluated for both influences on chromatographic separation conditions and MS sensitivity of target analytes. Formic acid (0.05–0.1 v%) caused significant signal suppression, particularly for BPA, OP, and NP such that the use of formic acid in the mobile phase was not feasible. Even when formic acid or acetic acid was only present in sample extracts and not removed completely during the drying steps, the signal suppression was observed. Also, traces of formic acid present in the LC-MS/MS system from prior analysis had to be flushed for a minimum of two days to avoid loss of MS sensitivity. The lack of flushing of the LC-MS/MS system is likely a significant source of the variable results observed in the literature, and it is critical to obtaining consistently low detection limits. Other common additives, including ammonia (1 v%), did not improve the MS sensitivity. Ammonium acetate (5–10 mM) slightly improved the LOQs for equilin (Eq) and PRNL, but increased the LOQs for the other compounds significantly, and thus was not used.

### 2.3. Matrix Effects

Initial evaluation of treated wastewater effluent and raw wastewater (0.1 mL sample extract diluted to 1 mL prior to analysis such that 50/50 *v/v*% acetonitrile/methanol) showed moderate to severe MS suppression. No significant difference in LOQs or linearity of the calibration curves were found when the matrix was present. Matrix interferences and baseline shifts were observed for the raw and treated wastewater samples, and more severe for the raw wastewater matrix. Recoveries for SPE and pre-concentration steps were >85% as assessed from the deuterated surrogate (4NP-d_4_ and EQ). For surface water samples, it is expected that the presence of humic and fulvic acids is the main attribute to the matrix suppression effect, and no chemical additives were added during the water treatment process.

To evaluate the influence of the matrix of the sample on the MS signal, the percentage matrix effect (%ME) was determined by comparison of the slope of the standard addition curve (which represents solutions where the standard is directly added to the sample extract and consequently matrix is present) with the slope of the calibration curve from solvent-based standards where

(1) $$\%\mathrm{ME}=\frac{\left(Slope\;of\;the\;standard\;addition\;curve-slope\;of\;solvent-based\;standards\right)}{Slope\;of\;solvent-based\;standards\;calibration\;curve}\;\times \;100.$$

For the purpose of the %ME calculation, an internal standard was not used in the calibration curves as its response might also be altered by the sample matrix to a different extent than the target analyte. Herein, we did not use a matrix-matched calibration curve as the matrix of the water samples from the wastewater treatment plant (WWTP)s was highly variable in location and time of sampling such that a representative matrix for the WWTPs was not feasible. A negative %ME value represented the MS signal suppression, and positive %ME values indicated MS signal enhancement. The categorization of matrix effect could be interpreted as soft (0 to ±20%), moderate (>±20 to ±50%), and severe (>±50%) [47]. If matrix effects were soft, then solvent-based calibration standards with internal standard calibration could be used for quantitative analyses (herein referred to as solvent-based standards). For moderate matrix effects, the matrix-matched standards or standard addition calibration were typically used, while for severe matrix effects, the standard addition was used if good linearity in the calibration curve and the required method limits of the quantitation could be obtained.

**Figure 3:**
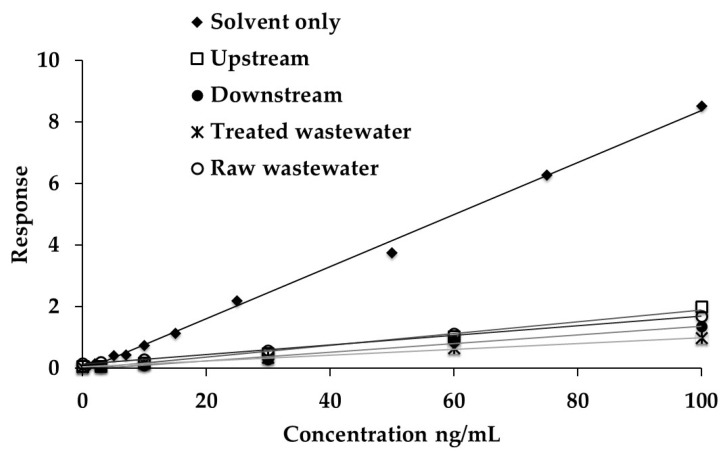
Calibration curves for BPA in the presence or absence of matrix from samples collected at a Wastewater Treatment Plant.

Figure 3 represents an example of the calibration curves (solvent-based and standard addition calibration) for BPA using the matrix from samples obtained at one WWTP. For the standard addition calibration, a calibration curve was completed for every sample and consequently, there was a standard addition calibration curve for the upstream, downstream, raw, and treated wastewater for each location. As can be seen, good linearity was obtained for the analytes for all calibration curves; however, the MS signal suppression was evident by the reduction in the slope of the standard addition calibration curves relative to the solvent-based (solvent-only) calibration. The calibration curves for BPA were typical of most analytes where the MS signal suppression was observed for all sample types (upstream, downstream, raw, and treated wastewater). All the water samples from the WWTPs showed a similar magnitude of matrix effect with the highest effect (either suppression or enhancement) in the raw wastewater collected at WWTP1, 4, and 5, and in treated wastewater collected at WWTP2 and 3.

**Figure 4:**
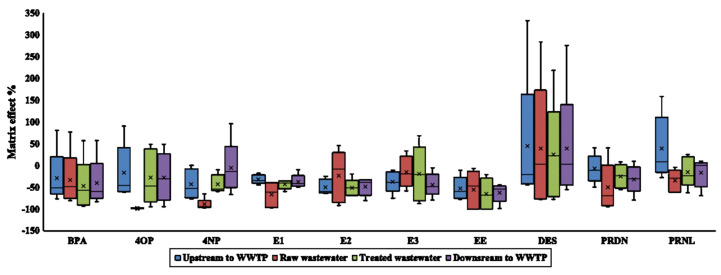
Percentage matrix effect for target analytes at Wastewater Treatment Plants in Saskatchewan. The average and median are represented by X and midline, respectively. Upper and lower box borders are the first and third quartile, respectively. The whiskers are minimum and maximum values.

Figure 4 shows the average matrix effects observed for each target analyte determined from water samples collected at five wastewater treatment plants located in Saskatchewan. The MS signal was suppressed for BPA, EE, and PRDN with a matrix effect generally ranging from moderate to severe in all the sample matrices. The matrix effect for 4OP and 4NP ranged from soft signal suppression or enhancement to severe signal suppression (>−50% ME) in treated and surface water. Severe MS signal suppression of 4OP and 4NP was observed for all raw wastewater sample matrices with a smaller variation in magnitude of %ME between sites. The MS signal at SRM (359→329) measured for PRNL was less prone to signal suppression than the signal at SRM (358→327) measured for PRDN. For both PRDN and PRNL, the magnitude of the signal suppression correlated with the expected complexity of the matrix in the raw, treated wastewater, upstream, and downstream surface water. The range of %ME for E1, E2, and DES was larger in the raw wastewater than other sample matrices attributed to the highly variable nature of the raw wastewater matrix at the different WWTPs. The response of DES at SRM (267→252) more commonly observed MS signal enhancement than other target analytes even in the raw wastewater, and there was evidence of the magnitude of %ME for a target analyte varying with the site location and SRM of each analyte. Baseline shifts in the chromatograms at and near the retention time of the DES were observed with some sample injections. DES was not detected in the surface or wastewater samples.

The largest average MS signal suppression was observed for 4OP in raw wastewater (99 ± 1.1%) at SRM 205 > 106. All other matrices had lower %ME with higher variability in %ME for the same transition of 4OP. Generally, soft to moderate matrix suppression was observed for natural estrogens in surface water at WWTPs located in larger population cities (WWTP1 and 2). Compounds with high retention times such as 4OP and 4NP commonly observed more severe matrix suppression in raw wastewater at all sites (average %ME was −100%). The average %ME for all WWTPs decreased (more MS signal suppression) with the matrix complexity (−19 ± 35%, −29 ± 29%, −31 ± 25% and −42 ± 39% for upstream surface water, downstream surface water, treated, and raw wastewater, respectively).

### 2.4. Concentrations of the Target Analytes

The new method involves the use of filtration followed by sample clean-up and pre-concentration with the C18 SPE and subsequent analyses of extracts from SPE using the developed liquid chromatography-negative ion electrospray ionization-tandem mass spectrometry (LC-ESI^-^-MS/MS) method which had an additive-free mobile phase. The application of this new method to the analyses of the target analytes in upstream, downstream, and raw and treated wastewater at five WWTPs in Saskatchewan was evaluated. As the matrix effects varied from soft to severe, the quantitative analysis was completed with standard addition calibration, and good linearity was obtained (R^2^ > 0.94, see supplementary Materials, Table S1; sample chromatogram shows BPA in wastewater compared to standard, Figure S2). The recoveries for SPE and pre-concentration steps were >85% as assessed from the deuterated surrogate (4NP-d_4_ and EQ) for all samples analyzed, which is a significant improvement as compared to other methods where recoveries could be <15% for surface water [1]. All the samples were obtained by WSA from the WWTPs, processed through the SPE sampling loading stage within 24 h, followed by completing the SPE, drying step, and LC-ESI^-^-MS/MS within five days to ensure the stability of the target analytes. Duplicate samples were collected at approximately the same time for each location. The % relative standard deviation (%RSD) of the duplicates was <10% in all the samples except in a few cases of the more difficult target analytes in raw wastewater samples such as E3 (%RSD 3–22.5%) and PRDN (%RSD 4–19%).

The frequency of detection of target analytes was low for surface water samples collected at upstream or downstream sites near the WWTPs in Saskatchewan as compared to wastewater collected from the WWTPs. Only selected target analytes were detected above the LOQ in surface water samples downstream of WWTPs, including BPA (11.8 ± 0.8 ng/L at one location) and 4NP (15.1 ± 1.7 ng/L at one location). E2 was also detected in an upstream surface water sample at one location (60.2 ± 2.6 ng/L). No other analytes were detected in surface water upstream to the WWTPs. The presence of E2 upstream could be due to a non-point agricultural source contributing to runoff to the upstream sample collection to this WWTP as there was no detection of E2 downstream to the same location (LOQ for E2 is 5 ng/L).

BPA was the most frequently detected xenoestrogen in raw wastewater (100% of samples) and treated wastewater (80% of samples), and the concentrations of BPA ranged from 133.3 ± 12.4 ng/L to 403.0 ± 16.1 ng/L in raw wastewater, and the concentration range was lower (<LOQ −175.5 ± 6.8 ng/L) in the treated wastewater. The maximum concentration of BPA was low relative to influent at other Canadian WWTPs [22]. Concentrations of BPA in the effluent (treated wastewater) are generally significantly lower than raw wastewater at other WWTPs worldwide as was measured at the WWTPs in this study. The presence of BPA has been reported in wastewater when the WWTP process uses a UV lamp, which is a common summer practice for the WWTPs in Saskatchewan [11]. The highest concentration of 4OP in raw wastewater was 305.8 ± 17.1 ng/L and at this WWTP 4OP was not detected in treated wastewater, as well 4NP was not detected in raw or treated wastewater. At one other WWTP, both 4OP and 4NP were detected in both raw and treated wastewater (132.9 ± 8.7 ng/L and 49.6 ng/L ± 7.4 ng/L in raw wastewater; 110.6 ± 5.8 ng/L and 57.9 ± 6.9 ng/L, respectively, in treated wastewater).

Detection frequency of E1, E2, and E3 in raw wastewater samples at the five WWTPs was 80%, 60%, and 100%, respectively. E3, E1, and E2 were detected in raw wastewater at all WWTPs with concentrations ranging from 21.4 ± 1.5 ng/L to 273.2 ± 16.2 ng/L, <LOQ to 156.0 ± 1.6 ng/L, and <LOQ to 99.1 ± 1.1 ng/L, respectively. At some WWTPs, the concentration of E1 was higher than E3 in raw wastewater, while at other WWTPs, the concentration of E3 was higher than E1. The concentration of E3 is expected to be a function of temperature, pH and photolysis rate and faster conversion of E1 to E3 may have occurred at some WWTPs. E3 is a degradation product of both E1 and E2, and the frequency of detection of E2 was lower than E1. E3 was detected in treated wastewater at three WWTPs from 12.0 ± 1.3 ng/L to 30.1 ± 0.8 ng/L. In treated wastewater, E1 was detected at one WWTP at 12.5 ± 1.5 ng/L, while E2 was detected at two WWTPs from (30.5 ± 0.1 ng/L and 107.0 ± 8.9 ng/L), which was within the range reported at other WWTPs [4,11,12,13,14,15,16]. Concentrations of E2 were significantly higher in treated wastewater than raw wastewater at one WWTP (107.0 ± 8.9 ng/L and 42.4 ± 4.7 ng/L, respectively), while other WWTPs in Saskatchewan observed lower concentrations of E2 in treated wastewater. Most WWTPs outside of Saskatchewan observed lower concentrations of E2 in treated wastewater than raw wastewater [4,11,12,13,14,15,16]. The higher concentrations of E2 in treated wastewater may have been due to the de-glucuronidation and de-sulfation of the conjugated E2 as suggested by a previous Canadian study [15]. The maximum concentration of E2 in treated wastewater in this study was higher than previously reported in the literature [13]. DES was not detected.

Prednisone (PRDN) is an analyte that is not commonly monitored in water or wastewater and was detected in raw wastewater collected from three of the five WWTPs (198.3 ± 14.2, 296.2 ± 56.7 L, 350.2 ± 14.3 ng/L), while prednisolone (PRNL), which is the degradation product of PRDN was only detected at one WWTP (PRNL in raw wastewater at 18.3 ± 0.2 ng and in treated wastewater at 57.8 ± 2.4 ng/L). PRDN was also detected in raw wastewater at this wastewater treatment plant, indicating that degradation can occur within the wastewater treatment process. Both PRDN and PRNL were not detected downstream, indicating that they were removed by the wastewater treatment process and PRNL has been shown to degrade by UV/chlorination process [48]. Concentrations reported for PRDN, and PRNL herein were lower than in wastewater influent of the WWTP in Switzerland [5].

## 3. Materials and Methods

### 3.1. Chemicals and Materials

All organic solvents (acetonitrile, ethyl acetate, methanol, and 2-propanol) are pesticide-grade analytical solvents supplied by Fisher Scientific (Ottawa, ON, Canada). Water for SPE and LC-MS/MS was distilled-deionized water (18 MΩcm resistivity) and was prepared in the lab using a Nanopure Diamond^TM^ System (Barnstead International, Dubuque, IA, USA). Aqueous ammonia (21% *w/v*) and glacial acetic acid (EMD chemical Inc.) were purchased from VWR Scientific (West Chester, PA, USA). The SPE format cartridges of Oasis HLB, MAX, and MCX (500 mg, 6 mL) were purchased from Waters Limited (Milford, MA, USA). C18 SPE cartridges (1000 mg, 6 mL) were purchased from Canadian Life Science (Peterborough, ON, Canada). All qualitative filter papers (glass microfiber filters 934-AH^TM^ and Filter papers 113^TM^) were purchased from Whatman (Florham Park, NJ, USA).

Standards of bisphenol-A, BPA, (97.0%), 4-octylphenol, 4OP, (99.0%), and diethylstilbestrol, DES, (99.6%), were purchased in solid form from Sigma-Aldrich (Oakville, ON, Canada), while 4-nonylphenol, 4NP, (99%), in solid form, was obtained from Fluka (St. Louis, MO, USA). Other natural and synthetic hormonal standards were obtained in solution. Estrone (E1), 17β- estradiol (E2), estriol (E3) and 17α-ethinyl estradiol (EE) (>90% purity) at 1.0 mg/mL standards in methanol (MeOH) were purchased from Cerilliant (Round Rock, TX, USA). Prednisone (PRDN) and prednisolone (PRNL), (>90% purity) 100 μg/mL in acetonitrile (MeCN) were supplied by Cerilliant (Round Rock, TX, USA). Bisphenol A-d_16_, (BPA-d_16_, 99.0%D), 4-nonylphenol 2,3,5,6-d_4_, (4-NPd_4_, (98.9%D) and gemfibrozil-d_6_ (2,2-dimethyl-d_6_) were purchased from C/D/N isotopes (Pointe-Claire, QC, Canada) in solid forms. Estrone 2,4,16,16-d_4_, (E1-d_4_, 97%D) was obtained in liquid form (5 mg/100 mL) from Cambridge isotopes laboratories (Tewksbury, MA, USA).

### 3.2. Sample Collection and Filtration

Pre-cleaned amber bottles were used for the collection of 1 L water samples and sampling was conducted by the Water Security Agency (WSA) at five wastewater treatment plants (WWTPs) in Saskatchewan. Additional samples upstream and downstream to each WWTP were also collected by WSA. The source of wastewater to the WWTP is mixed with sources including sanitary, industrial, and some agricultural discharge. All samples were delivered the same day of sampling and the filtration and solid-phase extraction (SPE) were completed within 24 h in a class 100 cleanroom. Immediately after delivery the water samples were filtered by vacuum filtration through glass fiber filters 934-AH (1.5 μm pore size) for the upstream, downstream, and treated wastewater samples. In most cases, raw wastewater samples had high particle loads and were first filtered through a Whatman 113 filter (30 μm pore size) prior to the 934AH filter to improve the speed of filtration.

### 3.3. SPE Sorbent Evaluations

Several sorbents for SPE were evaluated, including C18, Oasis HLB, Oasis MAX. After filtration, the pH was measured and adjusted to pH of 7.0 ± 0.1 using either acetic acid or ammonium hydroxide when C18 was the SPE sorbent. When the SPE sorbent was Oasis HLB or Oasis MAX the pH of the filtered water sample was adjusted to 3.00, according to prior developed methods [42,43]. To improve the flow through SPE sorbents 10 mL of methanol was also added per 1 L water. C18 SPE (or other SPE sorbents) were conditioned with 5 mL of ethyl acetate, 10 mL of methanol, and 10 mL of H_2_O at pH = 7 for C18, and pH = 3.0 for Oasis HLB or Oasis MAX. For testing of recoveries on these three sorbents, selected target analytes (BPA, 4OP, 4NP, E2, EE) were dissolved into 100 mL H_2_O and loaded onto the sorbents. Oasis HLB and C18 sorbents were washed with water and target analytes eluted with acetonitrile, while for Oasis MAX a wash solvent of 5 v% NH_4_OH (aq) and elution solvent of 2 v% formic acid in acetonitrile was selected. The volume of the wash solvent and elution solvent were optimized.

### 3.4. Solid-Phase Extraction

For water samples collected from upstream or downstream locations from the WWTP, and for treated wastewater, a 1 L filtered water sample with pH adjusted for the selected sorbent was used. For raw wastewater, a 500 mL filtered water sample was used. All samples were loaded onto the SPE sorbents at a rate of 200 mL/hour. Drying of the SPE cartridges was completed using a slight vacuum on the SPE manifold until the water was removed and then the SPE cartridge was stored in the dark at −4 °C. To ensure the stability of the analytes the filtration and loading of sample to the SPE sorbents were completed within 24 h. In addition, the elution of the target analytes from these stored SPE tubes, subsequent pre-concentration of the eluted extract, and LC-ESI^-^-MS/MS analysis was completed in five days or less. Standard tests showed that the target analytes were stable under these conditions.

For C18 SPE tubes were brought to room temperature and washed with 2 mL of water at pH = 7. Two surrogates (50 μL of 10 μg/mL 4NP-d_4_ and equilin at 500 ng/mL) were added and the wash was collected into F0. An additional 0.8 mL of acetonitrile (MeCN) was added to the SPE tube and eluted in the F0 fraction, which did not contain target pesticides. A 15.2 mL volume of MeCN was used to elute the target analytes into a 15 mL vial, which was labeled as the F1 fraction. The eluted F1 extracts were dried to 0.8 mL at a rate of 0.5 mL/hour in a SPE apparatus under slight vacuum (0.5 mmHg) and air flow (cleanroom air) from the slight opening of the SPE valves. After drying, the volume check was added at 25 μL of 10 μg/mL (250 ng/mL).

### 3.5. Apparatus and LC-ESI^-^-MS/MS Conditions

Instrumental analysis was conducted using a Waters LC system with 1525 μ binary pump, equipped with an autosampler from Leap Technologies (Minneapolis, MN, USA). The separation column was Phenomenex Gemini C18 (150 mm length × 2.0 mm i.d., 3μm particle size, pore size 100 Å) connected to a pre-column Phenomenex Gemini C18 (4 mm length × 2.0 mm i.d.). The flow rate was 0.2 mL/min and the injection volume was 5 μL. The mobile phase gradient used was 70/30 v% methanol/water (no additives) at time 0 min and increased linearly to 85 v% methanol from 0.00 to 4.00 min, held at 85 v% methanol for 3 min; increased to 100 v% methanol from 9.00 to 14.50 min. The re-equilibration of the column to the initial mobile phase took place for 10.10 min such that the total run time was 25 min. The target analytes eluted within 14 min.

The LC system is connected to the Waters Mircomass Quattro premier tandem mass spectrometry (Milford, MA, USA). The software includes MassLynx for instrument control and QuanLynx for data processing. The source temperature is set to 120 °C and the desolvation temperature was 390 °C. The desolvation N_2_ gas flow was 800 L/h, and the cone N_2_ gas flow was 100 L/h. Electrospray ionization was used in negative ion mode with the capillary voltage set to 2.90 kV. The collision gas was argon at 0.18 mL/min or 1.6 × 10^−3^ mbar. Infusion experiments were carried out in ESI^-^ mode using pure standards of each target analyte at 1 μg/mL and a syringe pump infusion rate of 50 μL/min. This was used to determine the SRMs and optimal cone and collision energy. SRMs for internal and surrogate standards were as follows: BPA-d_16_ (241→223, 241→241); E1-d_4_ (273→147, 273→145); 4NP-d_4_ (223→110, 223→223); equilin (268→143, 268→224); gemfibrozil-d_6_ (255→121, 255→255) and retention times were 5.44, 6.84, 13.80, 6.84, and 11.58 min, respectively.

## 4. Conclusions

A new sample preparation method was developed that used C18 SPE (1000 mg) for the pre-concentration of the target analytes from surface and wastewater samples. Target analytes more prone to stability issues included bisphenol A, 4-octylphenol and 4-nonylphenol. Strong retention on C18 SPE was also shown for 4-octylphenol and 4-nonylphenol. To reduce the elution volume required to elute the target analytes from the SPE sorbent and to improve their stability over the time required for analyses, the elution solvent was selected to be acetonitrile with no chemical additives. The presence of additives such as formic acid or ammonia in the sample preparation or mobile phase used for the separation greatly reduced the MS sensitivity. For all target analytes (diethylstilbestrol, 17β-estradiol, estriol, estrone, 17α-ethinylestradiol, prednisone, and prednisolone) negative ion mode electrospray ionization was used for detection in selected reaction monitoring mode with one transition used for quantitation and an addition transition used for confirmation. Even though the matrix effects were often determined to be moderate to severe for all sample matrices, a linear standard addition calibration curve could be obtained with similar LOQs to solvent-based calibration. The LC-ESI^-^-MS/MS method was demonstrated to be reliable for the detection and quantitation of the target analytes.

## Figures and Tables

**Table 1:** LC-ESI^-^-MS/MS parameters and recoveries, and limits of the quantitation method.

Analyte ^1^	Retention Time (min)	Quantitative SRM, Confirmation SRMs	Recovery% at 20 ng/L, 100 ng/L and 500 ng/L ±RSD% (n = 4)	SRM1/SRM2 Ratio (±RSD%) SRM1/SRM3 * SRM1/SRM4 **	LOQs (ng/L)
DES	6.57	267→252, 267→222, 267→237	86.0 ± 5.42, 87.4 ± 3.10, 95.3 ± 7.71	1.05 ± 6.52% 1.081 ± 7.29% *	5.0
E1	7.02	269→145, 269→143, 269→159, 269→183	88.0 ± 12.8, 93.6 ± 4.10, 98.3 ± 5.45	9.03 ± 19.9% 5.27 ± 23.1% * 8.30 ± 19.4% **	2.0
E2	6.95	271→145, 271→183, 271→271	89.7 ± 6.55, 105 ± 6.48, 105 ± 1.39	1.20 ± 8.58% 2.98 ± 10.5% *	5.0
E3	3.98	287→145, 187→171	82.4 ± 12.4, 102 ± 9.08, 98.3 ± 5.46	0.59 ± 21.5%	3.0
EE	6.69	295→145, 295→195, 295→295	99.8 ± 14.4, 112 ± 14.2, 108 ± 2.14	1.45 ± 21.3% 0.92 ± 10.6% *	10
BPA	5.62	227→212, 227→133	89.7 ± 4.23, 100 ± 3.84, 112 ± 3.28	1.56 ± 17.3%	1.0
4OP	13.13	205→106, 205→205	85.6 ± 7.98, 109 ± 10.3, 91.2 ± 8.18	1.00 ± 1.02%	1.0
4NP	13.80	219→106, 219→219	80.5 ± 9.49, 105 ± 13.0, 93.3 ± 5.88	1.00 ± 1.22%	2.0
PRDN	4.34	358→327, 327→300, 327→327,	84.3 ± 5.53, 96.9 ± 8.10, 90.0 ± 4.56	4.19 ± 9.27% 13.8 ± 14.5% *	10
PRNL	5.02	359→329, 359→259, 359→359	80.2 ± 6.56, 91.1 ± 8.14, 88.5 ± 15.0	3.82 ± 1.97% 10.86 ± 4.45% *	1

For analytes with more than two sensitive selective reaction monitoring transitions (SRMs) * notes ratio response SRM1/SRM3 and ** notes SRM1/SRM4 where SRM1 is the most sensitive SRM (SRM1); SRM3 is the 3rd most sensitive SRM; and SRM4 is the 4th most sensitive SRM. ^1^ See Section 3.1 for listed analytes abbreviations.

## Supplementary Materials

The following are available online. Figure S1: Quantitative and confirmation SRMs chromatographs of EDCs and internal and surrogate standards, Figure S2: The selected reaction monitoring chromatograms for BPA and internal standard BPA-d_16_ for raw and treated wastewater in comparison with similar levels of standard, Table S1: Linear regression equation of the best-fit curve and the R^2^ values for all the analytes from WWTP3 (for quantitative SRMs).
