# Supplementary material for: Determination of Endocrine Disrupting Chemicals in Water and Wastewater Samples by Liquid Chromatography-Negative Ion Electrospray Ionization-Tandem Mass Spectrometry
Source: Molecules. 2020 Aug 27;25(17):3906. doi: 10.3390/molecules25173906 (PMC7503312; doi:10.3390/molecules25173906)
Supplement: Supplementary file 1 [file molecules-25-03906-s001.pdf]

molecules

MDPI

Supplementary Material

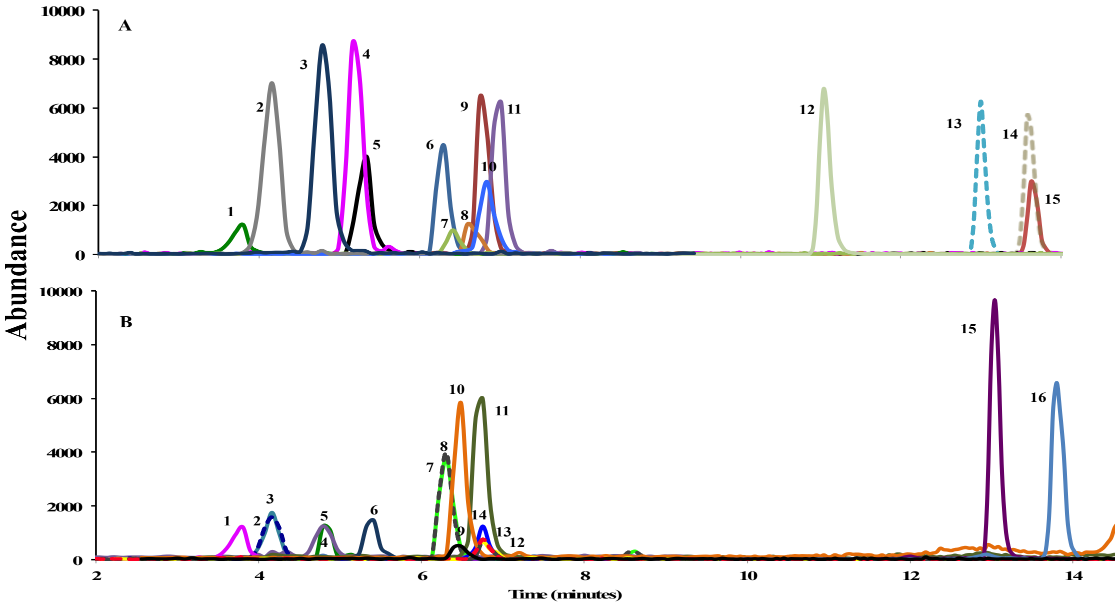

Figure S1. Quantitative and confirmation SRMs chromatographs of target EDCs and internal and surrogate standards. A, Quantitative SRM chromatographs with compound identity noted as follows: 1: E3 (287→145), 2:PRDN (358→327), 3:PRNL (359→329), 4: BPA-d_{16}(241→227), 5:BPA(227→212), 6:DES(267→252), 7:EE(295→145), 8:E2(271→145), 9:E1(269→145), 10:Eq(268→143), 11:E1-d_4(273→147), 12:Gem-d_6/50(255→121), 13: 4OP/10(205→106), 14:4NP-d_4/10(223→110), 15:4NP/10(219→106)]. B, Confirmation SRM Chromatographs. Compounds identity and selected SRM noted as follows: 1, E3 (287→171); 2, PRDN (327→300); 3, PRDN (358→328); 4:PRNL (359→259); 5:PRDL/10(359→359) 6:BPA (227→133), 7:DES/20(267→237), 8:DES/20(267→222), 9:EE/10(295→295), 10:EE (295→195), 11:E2(271→183), 12:EE/10(271→271), 13:E1(269→143), 14:E1(269→183), 15:4OP/65(205→205), 15:4NP/40(219→219). For some transitions, the abundance (Y-scale response) is divided by factor of 10, 40, 50 or 65 to show response of all analytes in the chromatogram as noted after compound identity. SRM monitored noted in brackets.

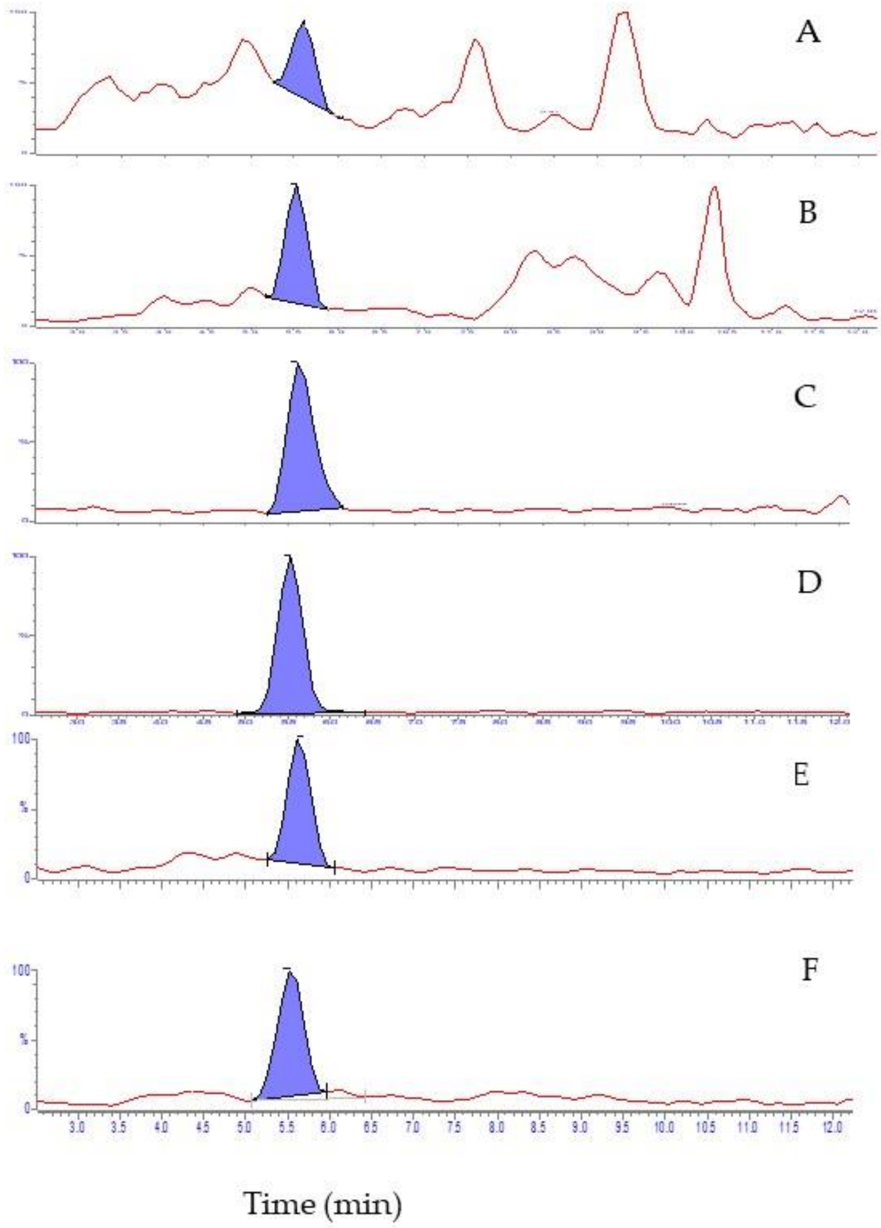

Figure S2. The selected reaction monitoring chromatograms for BPA and internal standard BPA-d_{16} for raw and treated wastewater in comparison with similar levels of standard. All chromatograms obtained on the same day of analysis. Chromatograms A, C, E are obtained at selected reaction monitoring transition 227→212 (quantitative SRM for BPA). Chromatograms B, C, F are obtained at selected reaction monitoring transition 241→227 (quantitative SRM for BPA-d_{16}). Sample matrix: Raw wastewater A and B; treated wastewater C and D; and 10 ng/mL BPA standard with BPA-d_{16} E and F. All samples contain BPA-d16 as an internal standard

molecules

MDPI

Table S1. Linear regression equation of the best- fit curve and the R^2 values for all the analytes from WWTP3 for quantitative SRM.

Analyte	Y=mx+b(r^2) Solvent-based calibration	Y=mx+b(r^2) standard addition with upstream water WWTP3 as matrix	Y=mx+b(r^2) standard addition with raw wastewater from WWTP3 as matrix	Y=mx+b(r^2) standard addition with treated wastewater from WWTP3 as matrix	Y=mx+b(r^2) standard addition with downstream water WWTP3 as matrix
BPA	Y=0.0846x-0.0877(0.997)	Y=0.0193x-0.0304(0.987)	Y=0.0156x+0.1285(0.997)	Y=0.0095x+0.0508(0.997)	Y=0.0142x-0.0877(0.997)
4OP	Y=0.5314x+0.4466(0.996)	Y=0.2108x+1.5498(0.997)	Y=0.0029x-0.0231(0.995)	Y=0.2725x+1.5498(0.991)	Y=0.3684x+0.2613(0.986)
4NP	Y=0.2175x+0.4182(0.990)	Y=0.1019x+0.0082(0.997)	Y=0.0105x+0.0329(0.992)	Y=0.1445x+0.4265(0.998)	Y=0.1511x+0.1252(0.999)
E1	Y=0.0272x-0.0228(0.999)	Y=0.0201x-0.0492(0.997)	Y=0.0174x-0.0709(0.973)	Y=0.0174x-0.0709(0.973)	Y=0.0158x-0.0286(0.990)
E2	Y=0.0015x+0.002(0.999)	Y=0.0009x+0.0039(0.998)	Y=0.0017x-0.0036(0.990)	Y=0.0005x+0.0033(0.940)	Y=0.0012x-0.0104(0.963)
E3	Y=0.0045x-0.0061(0.991)	Y=0.0011x+0.0008(0.982)	Y=0.0028x+0.0924(0.979)	Y=0.0009x-0.0076(0.985)	Y=0.0009x+0.0007(0.992)
EE	Y=0.0032x+0.0034(0.997)	Y=0.0007x+0.0011(0.982)	Y=0.0025x-0.0352(0.956)	Y=0.0021x-0.0368(0.992)	Y=0.0006x+0.0006(0.993)
DES	Y=0.0231x-0.0144(0.997)	Y=0.0994x+0.0568(0.999)	Y=0.0885x-0.0591(0.968)	Y=0.0736x+0.0012(0.996)	Y=0.0865x+0.0804(0.999)
PRDN	Y=0.0077x-0.0464(0.996)	Y=0.0108x-0.1122(0.995)	Y=0.0021x+0.0389(0.985)	Y=0.0058x-0.1241(0.980)	Y=0.0062x-0.0023(0.999)
PRNL	Y=0.0163x-0.0869(0.989)	Y=0.0421x-0.1217(0.992)	Y=0.0013x+0.0138(0.989)	Y=0.0304x+0.129(0.996)	Y=0.0178x-0.0149(0.990)
